# Conditional Similarity Triplets Enable Covariate-Informed Representations of Single-Cell Data

**DOI:** 10.21203/rs.3.rs-4915088/v1

**Published:** 2024-09-12

**Authors:** Chi-Jane Chen, Haidong Yi, Natalie Stanley

**Affiliations:** 1 Department of Computer Science, The University of North Carolina at Chapel Hill, Chapel Hill, NC 27599, USA; 2 Department of Computer Science, The University of North Carolina at Chapel Hill, Chapel Hill, NC 27599, USA; 3 Department of Computer Science and Computational Medicine Program, The University of North Carolina at Chapel Hill, Chapel Hill, NC 27599, USA

**Keywords:** single-cell, immune profiling, clinical prediction

## Abstract

Single-cell technologies enable comprehensive profiling of diverse immune cell-types through the measurement of multiple genes or proteins per cell. In order to translate data from immune profiling assays into powerful diagnostics, machine learning approaches are used to compute per-sample immunological summaries, or featurizations that can be used as inputs to models for outcomes of interest. Current supervised learning approaches for computing per-sample representations are optimized based only on the outcome variable to be predicted and do not take into account clinically-relevant covariates that are likely to also be measured. Here we expand the optimization problem to also take into account such additional patient covariates to directly inform the learned per-sample representations. To do this, we introduce CytoCoSet, a set-based encoding method, which formulates a loss function with an additional triplet term penalizing samples with similar covariates from having disparate embedding results in per-sample representations. Overall, incorporating clinical covariates leads to improved prediction of clinical phenotypes.

## 1 Introduction

High-throughput single-cell technologies, such as flow and mass cytometry and single-cell RNA sequencing are prominent experimental technologies for unraveling key genetic programs driving cellular heterogeneity [1,2]. In doing so, it is common to measure the expression of ~ 20–45 protein markers in each cell [3] to ultimately differentiate cells into specialized cell-types [4] and to characterize their functional responses. A range of recent and highly translational studies have illuminated characteristic changes in certain cell-types that are associated with certain disease states [5]. For example, there were shown to be certain chronological changes 1in frequencies of particular cell-types in the immune system that occur during a healthy pregnancy [6]. In this pregnancy example, as well as in numerous other applications [7–10], changes in frequencies or relative abundances that are associated with particular clinical phenotypes can be helpful for building predictive models of diseases or dynamic processes. Similarly, clinically-associated functional changes, such as the activation of particular signaling pathways, across cell-types can also be interrogated through single-cell immune profiling techniques. For example, a recent study uncovered specific cell signaling mechanisms that were implicated as patients recovered from surgical trauma [11].

To accommodate the information-rich datasets generated by single-cell technologies, machine learning methods have become crucial for linking complex patterns in cell-type abundances and their associated gene expression programs to clinical or experimental phenotypes comprising large clinical cohorts. In formulating a concrete clinical prediction task, there are numerous approaches for specifying quality per-sample feature encodings to adequately summarize cellular correlates for the outcome of interest. Moreover, such computed per-sample feature encodings or *featurizations* must be information-rich enough to capture subtle patterns in between sample heterogeneity, and align with the assumption that feature encodings are on, on average, more similar between samples with the same clinical outcome (label) than those with different labels. Broadly, featurization approaches are implemented by using either 1) biologically-meaningful per-sample feature engineering or by 2) mathematically abstract, yet detailed feature encodings. Two examples of biologically-interpretable feature representations include frequency feature engineering [12] and CytoDX [13]. CytoDx is a method for predicting clinical outcomes from cytometry data in a gating-free manner [13], averaging per-cell predictions across the sample. This is opposed to on features computed based on gates (whether manual or cluster-based). As a key limitation, CytoDx only implements a mean pooling operation to aggregate information across cells to the sample-level. CytoSet learns embeddings for single-cell data by encoding each sample’s molecular profiles into fixed-length vectors using permutation-invariant neural networks. This ensures consistent representations and minimizes a loss function by preserving local (cell-based) and global (profile-based) similarities. Therefore, CytoSet ensures embeddings not only capture cell relationships but also consider sample-specific characteristics, yielding more informative and interpretable embeddings. A prominent *gating-based approach*, which focuses on engineering cluster-level features in each sample, involves clustering cells into populations and counting the number of cells in each cluster. However, cluster-based feature engineering strategies are sensitive to the number of clusters chosen and are subject to variability across clustering runs. Another class of methods for linking cellular are broadly classified as feature-learning approaches, whereby a mathematical abstraction is learned for each sample, but lacks biological interpretability. These methods include CellCNN [14], random Fourier-based featurization [15], and CytoSet [16]. CytoSet is a deep-learning-based approach for encoding complex patterns in multiple CyTOF samples, which considers the outcome of interest [16]. The CytoSet model trains on multiple subsamples cells from individual samples. Although CytoSet successfully predicts clinical outcomes by subsampling across all samples for training the model, some datasets have complex interplays between clinical covariates and the outcome of interest, making prediction more challenging. CellCNN is another gating-free method that combines permutation equivalent and pooling functions for CyTOF data. Both CytoSet and CellCNN allow max or mean pooling to aggregate per-cell representations into a single per-sample representation. In using a permutation equivalent function to ensure results are robust to any permuation of cells, CytoSet enables the stacking of multiple layers to more flexibly learn from the input data, including both single-cell data and clinical outcomes. However, CellCNN only builds one permutation equivalent layer. Moreover, this particular architecture comprised of a single layer would likely be limited in its capacity to handle the complexity of single-cell data. The last strategy for predicting single-cell clinical outcomes is based on computing pooled Random Fourier Features (RFF) in each sample [15]. The RFF featurization approach broadly constructs mathematically abstract features for each cell within a sample and then aggregates these values across cells to obtain a single vector representation per sample. While RFF representations specify encodings for each cell into a high-dimensional space, it fails to generate a sample-wise encoding that is nuanced enough to effectively separate samples by their respective labels.

While various featurization techniques can translate complex cellular landscape patterns into useful feature encodings, these approaches are all agnostic to the collection of clinical covariates that may be available for each sample and potentially confound the outcome to be predicted. To address these limitations, we propose CytoCoSet, a deep-learning-based model that involves additional patient covariates distinct from the label for predicting clinical outcomes from single-cell data. This enhanced, covariate-informed model enables sample-based feature representation learning, which accommodates diverse covariates for a more holistic, clinically-holistic summary of the immune system and the patient’s background health. Further, our innovation extends CytoSet [16], which treats a multi-sample single-cell dataset as sets, enabling set-based encoding using a permutation invariant network architecture [17]. As a motivating example of a clinical study where covariates should be taken in account in featurizing the immune system, Peterson et al. introduced an immune profiling dataset of cord blood samples collected throughout pregnancy [7]. Many of these pregnancies resulted in preterm birth. To understand the immunological correlates of preterm birth in these settings, the authors showed that it was useful to include additional clinical covariates about the mother, such as gestational age, preeclampsia status, and miscarriage history. Therefore, while the objective is to predict likelihood of preterm birth from immune cells in cord blood, there is currently no way to take these many covariates into account in the prediction task. Moreover, each covariate also has a different level of correlation to the outcome label, which should also be considered in the problem formulation.To enhance per-sample immunological summaries with covariates, we update the CytoSet loss function with an extra penalty term, enforcing close embeddings between covariate-aligned samples. Overall, the integration of diverse clinically relevant summaries has the potential to enhance the accuracy and diagnostic power of single-cell immune profiling data in clinical settings.

## 2 Methods

CytoCoSet is a deep-learning based feature learning approach for encoding complex patterns into sample-level feature encoding that is predictive of clinical outcome. As our innovation, CytoCoSet augments the traditional supervised per-sample representation learning problem to also take into account the rich set of patient-specific covariates that are likely driving between-sample variation and outcome. Briefly, the model is formulated as an extension to the set-based encoding method, CytoSet [16], which is optimized to find per-sample representations that are predictive of clinical outcome. CytoCoSet extends the per-sample feature learning approach by augmenting the original loss function with a triplet term enforcing similar learned embeddings between samples with similar covariates, inspired by the Conditional Similarity Triplets described in Ref. [18]. Moreover, the learned embeddings can identify novel sample subsets with similar immunological correlates of particular clinical outcomes. Triplets, consisting of three samples with varying similarities, enhance the loss function. We also propose an innovative strategy to select key triplets using Random Fourier Features. Key components of the algorithm, including the loss function, triplets selected by Random Fourier Features, and model architecture, are detailed in the following sections.

### Computing Random Fourier Feature Representations for Each Sample.

Specifying triplets involves selecting combinations of three samples, where two are similar and two are distinct based on a measured covariate. Here, we sought to specify triplets that were the most information-rich and would therefore have a strong effect on the loss. For example, we sought triplets, such that the pair of *different* (*similar*) samples are as different (similar) in terms of their overall immune profiles, as possible. We therefore developed an efficient, unsupervised approach to encode per-sample immunological landscapes to be able to infer between-sample similarities. Per-sample representations were computed with Random Fourier features [15]. Briefly, Random Fourier features infer abstract, mathematical d-dimensional feature encodings per cell, which can be averaged across all cells in a sample to create a per-sample encoding. In practice, the Random Fourier Feature representation for *i*th sample in Fig. 2 $z_{i}\in R^{m\times d}$ is computed by approximating the feature map of the Gaussian kernel. First, the input considers a ith sample’s m×n single-cell data matrix $A_{i}$, which encodes the expressions of n proteins in m cells. Columns of $A_{i}$ are first transformed based on a *d/*2-dimensional randomly generated normal random variables. So, for a given column j of $A_{i}$, we generate a transformation vector, $P_{j}$ of n normally distributed random variables, such that each entry of $P_{j}∼𝒩(0,\frac{1}{\gamma })$.

Moreover, the $P_{j}s$ can be concatenated row-wise to form an overall transformation matrix,<$P\in R^{n\times d/2}$ with $P=\left[P_{0}\left|P_{1}\right|\cdots |P_{n-1}\right]$. Ultimately, the input matrix $A_{i}$ is transformed to ${A^{\prime }}_{i}$ as,

(1) $${A^{\prime }}_{i}=A_{i}\times P.$$

Next, each row of ${A^{\prime }}_{i}$ is further transformed into a vector, such that the q th row of ${A^{\prime }}_{i}$ yields a vector, $z_{i}$ by applying sine and cosine evaluations to each element. That is, for the q row, ${A^{\prime }}_{iq}$, the vector $z_{i}$ is computed as,

(2) $$z_{i}=\sqrt{\frac{2}{K}}[\text{cos}\left({A^{\prime }}_{i,0}\right),\ldots ,\text{cos}({A^{\prime }}_{i,\frac{d}{2}-1}),\text{sin}({A^{\prime }}_{i,0}),\ldots ,\text{sin}({A^{\prime }}_{i,\frac{d}{2}-1})]$$

Here, K is the number of random features $\left(\frac{d}{2}\right)$ used so, $\sqrt{\frac{2}{K}}$ is negligible. Moreover, we take the individual $z_{i}s$ and concatenate them by row to form a matrix, $z^{i}$ of transformed representations across cells as,

(3) $$z^{i}=\left[\begin{array}{c} z_{0}\\ \cdots \\ z_{q-1} \end{array}\right],q=0,\ldots ,m-1$$

Finally, to compress the matrix $z^{i}$ (cell embedding per sample i) into a vector $S_{i}\in R^{1\times d}$, we apply a chosen pooling operation from one of several possibilities to each column of $z^{i}$ as,

(4) $$S_{i}=\left\{\begin{array}{l} {Pool}_{\text{median}}\left(z^{i}\right)\\ {Pool}_{max}\left(z^{i}\right). \end{array}\right.$$

Note that median and max pooling are common, and we found median pooling to be the most robust in our experiments.

Ultimately, these per-sample representations, $S_{i}$, are used to compare the aggregate immune landscape between pairs of samples.

### Selecting Triplets Based on Random Fourier Features.

**Fig. 1:**
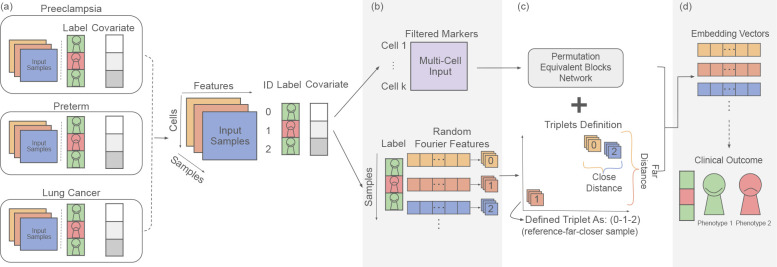
Overview. Schematic overview of CytoCoSet. (**a**)Given a multi-sample single-cell dataset with a sample-specific clinical outcome and additional measured sample covariates, (**b**)the CytoCoSet algorithm defines a set of triplets to constrain the process of learning per-sample embedding vectors, based on random fourier features. A triplet is a combination of three samples, such that two samples should have similar embeddings, and the third sample is different and should therefore have a more divergent embedding. (**c**) The loss function specified to optimize the embeddings is comprised of a binary cross entropy term to enforce prediction accuracy, and a triplet term, which encodes covariate-based similarity constraints. (**d**) Finally, embedding vectors learned by the model are trained to predict each sample’s clinical outcome.

**Fig. 2:**
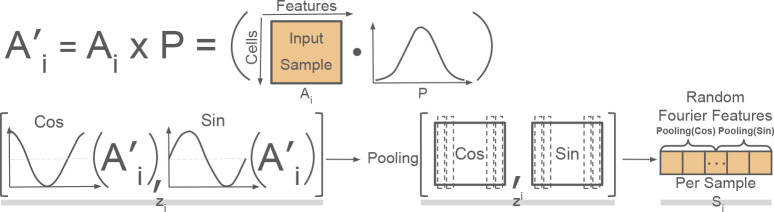
Overview of Random Fourier Features for Triplet Selection. An illustration of how Random Fourier features are used to summarize the overall immune landscape for each sample. The columns of a cell × feature input matrix $A_{i}$ is transformed with $\frac{d}{2}$ Gaussian random variables to produce a new matrix, ${A^{\prime }}_{i}$. Per-cell Random Fourier features are constructed by concatenating sine and cosine transformed values of ${A^{\prime }}_{i}$ to form a matrix $z^{i}$ across all cells. Finally, a Random Fourier Feature vector $S_{i}$ is constructed by pooling in each dimension.

Here, we describe the framework for using the Random Fourier Feature representations of samples to optimally improve model training. We define $S_{i}$ as the vector of Random Fourier features for sample i. Each triplet is comprised of three samples, namely with indices i, j, and k, and also with associated RFFs $S_{i}$, $S_{j}$, and $S_{k}$. This set of triplets is therefore comprised of a reference sample ($S_{i}$), a sample deemed to be distant from $S_{i}$, $S_{j}$, and a sample, $S_{k}$, that is similar to $S_{i}$. Note that we refer to this relationship between members of a triplet as reference sample-more distant sample-closer sample, throughout Fig. 1 (c). Moreover, given the Random Fourier feature representations of each sample, we can compute their pairwise similarity with Euclidean distance, given as $D\left(S_{i},S_{j}\right)=\sqrt{\sum_{x=1}^{d}\;{\left(S_{i}^{x}-S_{j}^{x}\right)}^{2}}$, where each $S_{i}^{x}$ denotes the x-th component of the RFF vector in sample i. We further applied two different rules for choosing triplets, with respect to selecting *same* and *different* members in relation to the reference, with hyperparameters $H_{s}$, and $H_{d}$. Hyperparameter $H_{s}$ controls the selection of the *same* triplet member, therefore giving the cutoff point for selecting a suitable $S_{k}$ deemed to be similar enough to the reference sample, $S_{i}$ according to the computed distance $D\left(S_{i},S_{k}\right)$. Alternatively, hyperparameter $H_{d}$ gives the threshold cutoff for a sample to be considered different from the reference, and is chosen according to a distance computed between the reference and the ‘different’ member of the triplet and the reference and the ‘same’ member of the triplet as,$D\left(S_{i},S_{j}\right)-D\left(S_{i},S_{k}\right)$. Ultimately, $H_{d}$ is a tuned to be some quartile across evaluations of D(⋅) on all triplets. In practice, paratmers $H_{s}$ and $H_{d}$ were tuned across possible quartiles in increments of 20, as evaluated in all triplets.

### CytoCoSet Loss function.

The loss function is comprised of two terms, which is used to optimize parameters in a manner which 1) lead to high classification accuracy, and 2) enforces embeddings to respect similarity patterns between members of the triplets. To promote classification accuracy, we used standard binary cross entropy, which quantifies the overlap between the true and predicted labels. Margin ranking loss was selected to quantify the preservation of the triplet information. Further, we specify a parameter, α to control the trade-off between each of these two components in the loss. Putting both components together, we seek to minimize the following loss, ℓ in equation 5 as,

(5) $$\ell =\alpha \frac{1}{B}\sum_{i=1}^{B}\;\underset{\text{Binary}\;\text{Cross}\;\text{Entropy}}{\underbrace{BCELoss\left(f_{\theta }\left(x_{i}\right),y_{i}\right)}}+(1-\alpha )\underset{\text{Triplet}\;\text{Similarity}}{\underbrace{T\left(S_{i},S_{j},S_{k}\right)}}.$$

Here, B denotes the batch size or the number of samples used in each batch of training, $f_{\theta }$ denotes the probabilities of particular sample label prediction within n measured proteins, and α denotes the balance between the two terms of the loss. The learning rate and batch size are set to 0.0001 and 200 across different datasets, respectively. In the binary cross entropy term, $x_{i}$ encodes the cell × feature matrix for sample i, and $y_{i}$ gives the binary clinical outcome label for the i-th sample.


(6) $$T\left(S_{i},S_{j},S_{k}\right)=max\left\{0,dist\left(S_{i},S_{k}\right)-dist\left(S_{i},S_{j}\right)+h\right\}$$

In the triplet loss term, $T\left(S_{i},S_{j},S_{k}\right)$ shown in equation 6, provides a penalty to prevent the learned embeddings from violating covariate-level information, enforced through the triplets. Intuitively, this term will add an ideal 0 to the loss if the embeddings for the reference and the *same* (*diff*) samples are more similar (different)). The parameter h is an offset parameter or margin to avoid trivial solutions. Specifically, the dist here is euclidean distance.

## 3 Results

We evaluated the performance of CytoCoSet along with several related methods on three CyTOF datasets. Our experiments explore outcome prediction accuracy, correlation of embedding distances with covariates, and usefulness of the many covariates that could be chosen.

### 3.1 Datasets

To explore the capacity of CytoCoSet to predict clinical outcome by integrating covariate information, we applied our model to three publicly available multi-sample CyTOF datasets, which we briefly introduce here.

#### Preeclampsia dataset.

The preeclampsia dataset [19] profiles 45 women throughout their pregnancies, where a subset of the women developed preeclampsia. There were 33 protein markers measured per cell. The clinical outcome to be predicted was whether the sample was from a preeclamptic or control sample. We further used gestational age, ranging between 8 and 28 weeks, as the covariate. **Preterm dataset.** The preterm CyTOF dataset [7] profiled cells extracted from cord blood in 42 women, where 17 women delivered at term and 25 women delivered prematurely. There were 36 protein markers measured per cell. Moreover, we sought to predict whether each sample was from a woman who delivered term or at preterm. We further considered several relevant covariates across different experiments (as our method can currently only handle a single covariate). These covariates include, gestational age, history of miscarriage, and preeclampsia status. **Lung cancer dataset.** The lung cancer dataset [20] collected samples from 27 patients, and measured 31 proteins per cell. We sought to predict whether or not each patient had progression-free survival (PFS). To formulate a classification problem, we binarized these continuous PFS measurements based on the median. We considered several available covariates (again, across separate experiments), including binarized age, sex, systemic immunosuppressive treatment for adverse events, and drug-related adverse events. Ultimately, we selected the administration of immunosuppressive treatment to be the dominant covariate to be included in analysis.

Next, we provide a brief discussion about how related works were implemented to enable comparison with CytoCoSet.

### 3.2 Related Methods Evaluated in Experiments

The experiments involved four related methods for converting single-cell profiles into immunological summaries, including, CytoSet [16], k-means with feature engineering [12], featurization via Random Fourier Featurs [15], and CellCNN [14]. We note that none of these methods take covariates into account, and hence are evaluating the overlap between the immune system and the clinical outcome.

#### CytoSet [16]

First, CytoSet is a deep learning-based method for learning representations for each sample that are predictive of clinical outcome.

#### k-means with feature engineering [12]

Under the k-means and feature engineering method, we cluster the cells, and then compute per-cluster frequencies, which reflect the proportion of each sample’s cells assigned to each cluster. Cluster frequencies are then input into a random forest classifier to predict the clinical outcome.

#### Random Fourier Features [15]

Random Fourier features (RFFs) are used to featurize each sample by projecting each cell in each sample into a higher dimensional space and then ultimately computing a per-sample representation by pooling the values of cells across each dimension (e.g. taking the max). RFFs can then be given to a classification model, here we applied Random Forest classifier, to predict outcomes.

#### CellCNN [14]

CellCNN is a single-cell analysis algorithm with a pooling layer after the convolutional layer combining the cell-level features to generate a single representation for the entire sample. By using a cross-entropy loss to measure the difference between the predicted probabilities and the true class labels or mean squared error to minimize regression problems, CellCNN output ensures the sample-level feature vector leads to an accurate prediction of clinical phenotype.

### 3.3 Predicting Clinical Labels Across Datasets

**Fig. 3:**
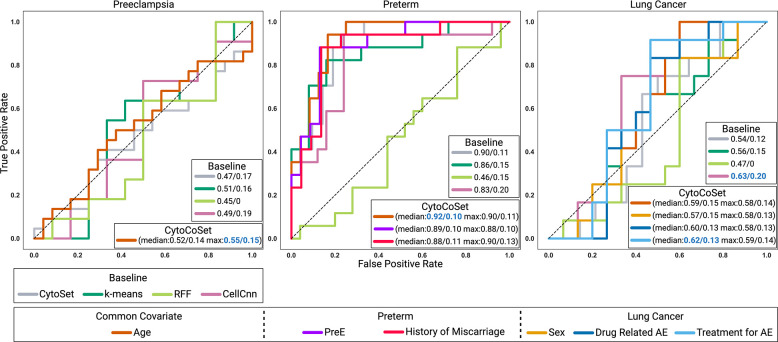
Classification Accuracy in CyTOF Datasets. CytoCoSet and baseline methods were evaluated for their capacity to produce per-sample encodings that could accurately predict a binary clinical outcome in three CyTOF (Preeclampsia, Preterm, Lung Cancer) datasets. All classification experiments were done using 30 different train/test splits, and ROC curves corresponding to the particular trial that achieved the mean AUC. The legend for each ROC curve shows the (mean/standard deviation) for various combinations of method and covariate. AUC results for median and max pooling strategies employed to compute Random Fourier Features for selecting triplets in CytoCoSet are also shown.

In our first set of experiments, we wished to predict the clinical labels of all samples, using CytoCoSet and related methods. In general, we sought to test our intended objective of whether incorporating covariates produces per-sample encodings that increase clinical outcome prediction accuracy. In these experiments, we focused on classification, and therefore binarized any continuous clinical outcomes or covariates based on their median. With all tasks being binary classification problems, area under the ROC curve (AUC) was used as the metric of success. ROC curves for classification tasks in each of the three datasets are shown in Fig. 3.

Across datasets, the performance patterns of the various methods revealed notable insights. For example, in the lung cancer dataset, CytoSet achieved an AUC of 0.54, and CytoCoSet was able to further improve on this accuracy using the systemic immunosuppressive treatment for adverse events as the covariate and achieving an AUC of 0.62. Alternatively, when using drug-related adverse events as the covariate, CytoCoSet achieved only marginally worse performance than that obtained using the systemic immunosuppressive treatment for adverse effects as the covariate. Finally, the performance of the model using age as the covariate achieved an AUC of 0.59, which is still stronger than CytoSet. While the CellCNN approach technically achieved the highest AUC, it also showed substantial standard deviation, suggesting a lack of robustness of such an approach. In the preeclampsia dataset, CytoCoSet achieved an AUC of 0.55 using binarized age as the covariate and max pooling in the RFF process, which surpassed CytoSet’s AUC of 0.47. In the preterm dataset, by construction, gestational age is highly correlated with the term of preterm status, so we anticipated the covariate would help performance. While CytoSet achieved an AUC of 0.9, CytoCoSet attained an AUC of 0.92. Conversely, the featurization via RFFs exhibited the lowest AUC of 0.46, followed by frequency feature engineering and CellCNN, which achieved AUCs of 0.86 and 0.83, respectively.

Across multiple datasets, various covariates exhibit differing degrees of effectiveness in enhancing prediction accuracy. For example, age is a common covariate across datasets, but it is not necessarily always helpful for clinical outcome prediction. Notably, in the preeclampsia dataset, the inclusion of age information yields a substantial increase in AUC. Conversely, in the preterm dataset, the impact of age as a covariate is comparatively modest, and only marginally increases AUC. The noteworthy enhancement of prediction accuracy with age as a covariate as observed in the preeclampsia dataset suggests that immune profiles augmented with age could produce a more accurate predictor of preeclampsia.

### 3.4 Evaluating Alignment of Embedding Vectors and Covariates

**Fig. 4:**
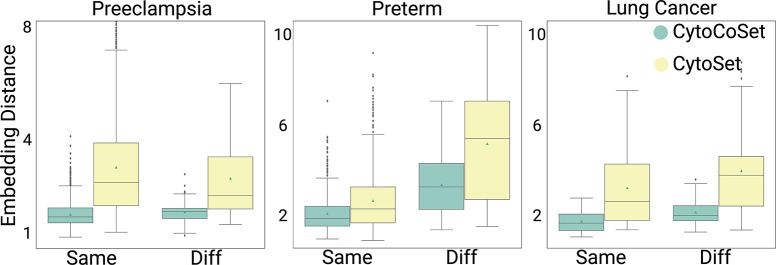
Quantifying Alignment of Embedding Vectors with Covariates. We visualized distances computed between pairs of samples with with same-age (denoted as ‘Same’) and those with different-age (denoted as ‘Diff’) under CytoSet (yellow) and CytoCoSet (green) approaches. The boxplots display pairwise distance distributions for each dataset and covariate matching condition, with a green triangle denoting the mean embedding vector distance.

We designed an experiment to evaluate how well the learned embedding vectors align with age. If CytoCoSet identifies age-aligned embeddings, vectors from samples of the same age should be most similar. To test this, embedding vectors were extracted in each sample based on the second-to-last layer of the network. For many random pairs of samples (specifically 66, 190, and 120, in the lung cancer, preeclampsia, and preterm datasets, respectively), we computed the Euclidean distance between their respective embeddings returned in the second-to-last layer. This experiment was repeated over thirty trials to generate a distribution of accuracies between pairs of samples that both had the ‘Same’ and ‘Diff’ (different) ages, using both CytoCoSet and CytoSet. These distributions are visualized with green and yellow boxplots in Fig. 4, respectively. We observed that the distances between the embeddings computed under CytoCoSet in the same category tended to be lower than the covariate-agnostic variant, CytoSet, across the three CyTOF datasets. In light of the varying quality in age covariates observed across the three datasets, we established dataset-specific thresholds for determining whether a pair of ages was deemed to be the same or different.

In the preterm dataset, same-age was defined as pairs with less than four weeks difference and different-age as greater than ten weeks. Conversely, within the lung cancer dataset, same-age was less than two years difference and different-age was more than thirty years. For the preeclampsia dataset, same-age was less than five weeks difference and different-age was more than eighteen weeks. Analyses of pairwise embedding vector distances across the *same* and *different* comparisons revealed consistent trends across the three datasets. Notably, CytoCoSet exhibited generally lower pairwise distances in both the same and different categories. Additionally, all observed trends, except CytoSet on the preeclampsia dataset, showed expected patterns of similar embedding vectors for same-age samples. In the preeclampsia dataset, CytoSet erroneously showed smaller embedding vector differences of sample pairs, whereas CytoCoSet correctly exhibited smaller pairwise embedding vector differences for same-age samples. This observation suggests that CytoCoSet offers an advantage in assuring that samples with the same covariate, in this case, age, have more similar learned embedding vectors.

### 3.5 Sensitivity of Parameters In the Loss Function

**Fig. 5:**
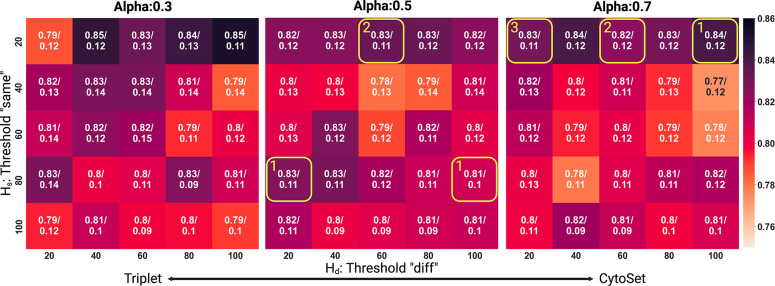
Sensitivity Analysis of Parameters within the Loss Function. We systematically varied the three model hyperparameters, α, ‘same threshold’ ($H_{s}$) and ‘diff threshold’ ($H_{d}$) and visualized the mean AUC/standard deviation over ten trials with different train/test splits, in a heatmap. The experiment demonstrates that a stringent ‘same’ threshold significantly influences the outcomes more than the ‘diff’ threshold. Ten trials were run with different train/test splits, and yellow squares show optimal hyperparameter combinations across the ten trials. Each heatmap grid also denotes the number of trials that a particular parameter combination was optimal in.

The tuneable parameters in the model include the ‘same threshold’, $H_{s}$ ‘diff threshold’, $H_{d}$ and α. In practice, lower quartile values for $H_{s}$ and $H_{d}$ involve more stringent triplets with closer ($S_{i},S_{k}$) distances and larger ($S_{i},S_{j}$) distances. Next, α, controls the relative contributions of the binary cross entropy term and the triplet term in the loss function. We systematically varied all three parameters and visualized the resulting mean/standard deviation of the test-set AUC, obtained across ten trials in the preterm dataset (Fig. 5). All experiments here constructed triplets through median pooling based on Random Fourier Features. This experiment revealed that having a stringent same threshold, $H_{s}$ impacts the results more than the diff threshold, $H_{d}$. That is, choosing triplets more appropriately for the same threshold will have a more productive effect on the loss. During ten trials in the CytoCoSet parameter tuning experiment, $H_{s}$ and $H_{d}$ were varied with α=0.3,0.5,0.7, obtaining optimal values for α=0.5 and α=0.7. Specifically, we denoted these hyperparameter combinations with yellow squares and reported the number of optimal parameter combinations for each trial in Fig. 5. Overall, CytoCoSet revealed some consistency in optimal parameter value, but there was still some variation.

### 3.6 Examining Effects of Different Covariates

**Fig. 6:**
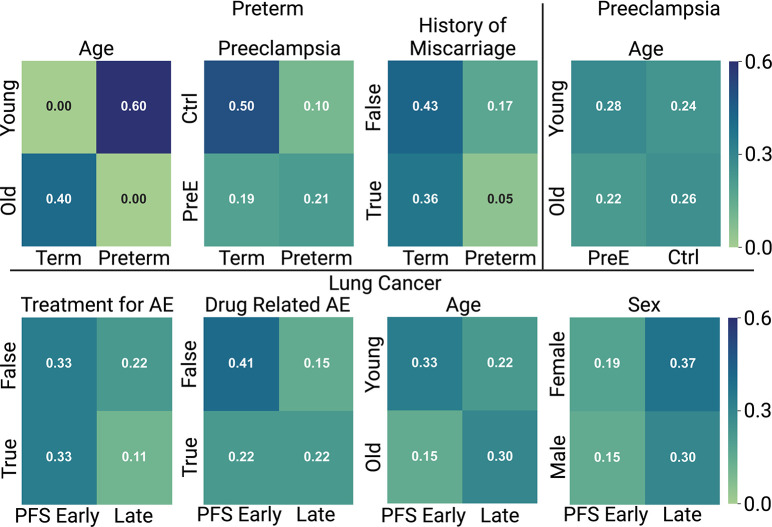
Variability in Covariate Quality for Learning Useful Embeddings. The heatmap shows the correlation between the label to be predicted and each of the possible covariates in each of the three datasets. Ideally, if a covariate is related to the outcome to be predicted, there will be high scores on the diagonal. Darker colors therefore indicate a strong relationship between a particular label and covariate. For example, in the preterm dataset there is an expected high correlation between the preterm (term) label and the young (old) covariate.

Each dataset typically includes multiple covariates per patient, which may or may not correlate with the outcome. Our assumption that embedding vectors should be more similar for samples with similar covariates is only valid if such covariates are aligned with the label used to optimize the network parameters. Here, we explored how varying covariates affected classification accuracy in the preterm and lung cancer datasets. Fig. 6 shows the correlation between the label to be predicted and each of the available covariates. In the preterm dataset, we observed age to be highly correlated with the preterm condition, indicating that this is a good candidate covariate to use to inform the embeddings. Alternatively, the history of miscarriage covariate did not seem to correspond to the preterm label. Similarly, we looked at the range of covariates in the lung cancer dataset, including, treatment for AE, drug related AE, age, and sex. None of these covariates seemed to have an extraordinarily high correlation with the PFS early or late condition. The heatmap of the correlation between the true label and each of the possible covariates in the lung cancer dataset is also shown in Fig. 6.

Moreover, we designed a statistic to efficiently identify which covariate is most associated with the outcome to be predicted and hence would be most helpful for model training. Given binary values for a particular covariate of interest and a binary outcome, we can construct a two-by-two square matrix representing the correlation between each binary outcome and each of the binary values of the covariate. Thus, the top left, top right, bottom left, and bottom right quadrants are denoted as $D_{0}$, $D_{1}$, $D_{2}$, and $D_{3}$, respectively. Given this constructed matrix, we can compute the statistic to quantify alignment between a covariate and an outcome as,

(7) $$\text{Covariate}\;\text{Dependency}=\left|\left(D_{1}+D_{2}\right)-\left(D_{0}+D_{3}\right)\right|$$

**Fig. 7:**
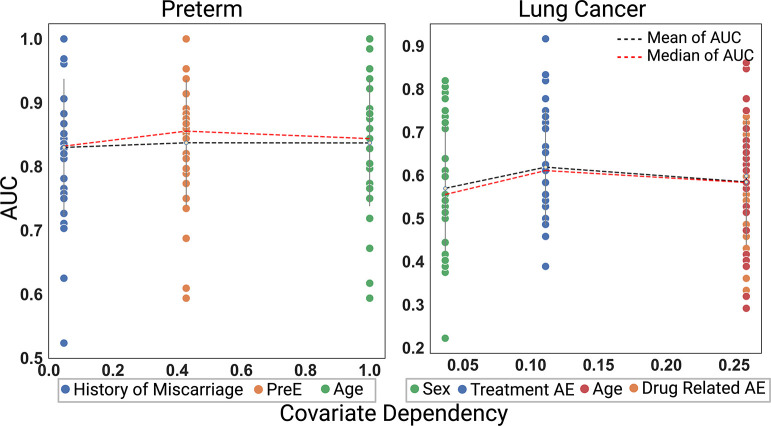
Evaluation of All Possible Covariates in the Preterm and Lung Cancer Datasets. We examined how the extent to which covariates align with the outcome to be predicted in the preterm and lung cancer datasets. Higher values of ‘covariate dependency’ (horizontal axis) imply stronger alignment between a covariate and the outcome. CytoCoSet was run over 30 trials, under each covariate. Here, covariates are ordered as a function of increasing covariate dependency score. We reported AUC (dots, vertical axis) as the metric of success to quantify how useful the embeddings were in the classification task.

This computed statistic, Covariate Dependency, captures the difference between two sums of the diagonal, which capture the difference between a given covariate level and an outcome. In an ideal setting, this difference would be large indicating strong alignment of an outcome with a particular covariate. We set up prediction tasks in each datasets under each possible covariates and plotted the distribution of AUCs (each trial represented by a dot) over 30 trials in Fig. 7 as a function of covariate dependency. We expect the AUC to rise as covariate dependency increases. Thus, a higher AUC is anticipated as the difference becomes more pronounced, indicating enhanced discriminative ability and predictive accuracy in the model. The results suggest that mean or median AUC (shown by dotted line) is generally larger for more informative covariates, or those with larger covariate dependency. Specifically, in the preterm experiment, the covariate ‘age’ exhibits the highest degree of significance, as evidenced by its high covariate dependency value, consistent with the findings illustrated in Fig. 3 pertaining to the age AUC within the preterm dataset. Within the lung cancer experiment similarly, ‘drug-related adverse events’ covariate has the highest covariate dependency, which also has a relatively high AUC in Fig. 3.

### 3.7 CytoCoSet Captures Between-Sample Variation

**Fig. 8:**
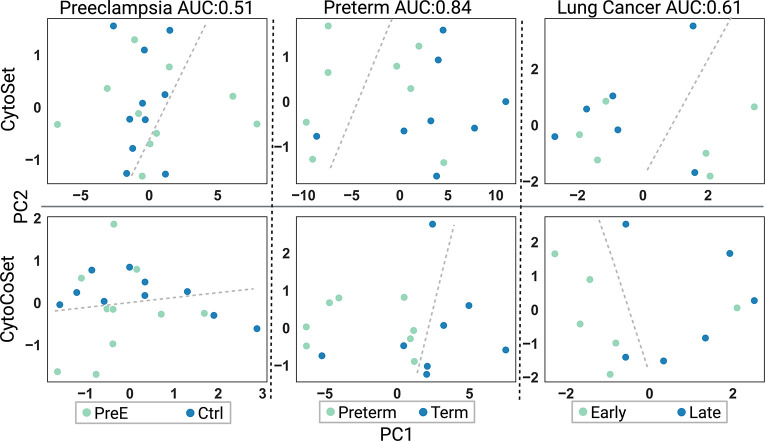
Visualizing Samples in 2d Based on Learned Embeddings. PCA was used to project samples in two-dimensions with PCA, based on the learned embedding coordinates under CytoSet (top) and CytoCoSet (bottom). Thirty classification trials were run and we visualized test set samples corresponding to the trial achieving the median AUC. Dots represent samples and are colored by the class label. Gray dotted lines represent a possible decision boundary that could be used to separate the samples correctly.

We sought to understand the extent to which the learned embeddings captured variation between samples. That is, we expected high-quality embeddings could be input to a classifier or dimension reduction method to adequately separate samples by the predicted outcome. We ran 30 classification trials with different train/test splits to predict outcome based on embedding vector and chose the trial resulting in the median AUC to visualize each profiled sample in two-dimensions (the first two principal components) with PCA. These results are shown in Fig. 8, and samples are colored by their respective outcomes. The gray dotted line shows a possible decision boundary that could separate points in different classes, as achieved using the Nu-Support Vector Classification. In the preeclampsia dataset, 20 samples were used in the test set, with age as the covariate, and overall achieved a median AUC score of 0.51. Using age as the covariate, CytoSet and CytoCoSet classified six and five samples incorrectly, respectively. In the preterm dataset, age was also used as the covariate, and achieved a median AUC score of 0.84 over the thirty trials. Sixteen samples were included in the test set, and five and three samples were classified incorrectly under the CytoSet and CytoCoSet approaches, respectively. In the lung cancer dataset, treatment for adverse events was used as the covariate, and achieved a median AUC score of 0.61 over the thirty trials. Twelve samples were included in the test set, and four and two samples were classified incorrectly under the CytoSet and CytoCoSet approaches, respectively.

## 4 Conclusion

The paper defines a robust method for incorporating patient-specific clinical covariates to learn per-sample featurizations to summarize each patient’s cellular landscape via a learned embedding vector. Our method, CytoCoSet, builds on CytoSet [16], a set-based encoding method to robustly take into account per-sample outcome labels to be predicted with additional patient covariates. As per-sample featurizations or encodings are often used in downstream predictive modeling tasks for linking cellular composition to a clinical phenotype, CytoCoSet addresses a fundamental limitation of current approaches, by leveraging both patient-level covariates and clinical outcomes in the training process. Specifically, we enhance the conventional set-based encoding formulation of the loss function proposed for learning model parameters with CytoSet by introducing a triplet term. Such a term penalizes learned embedding vectors from being substantially different between samples having similar covariates. CytoCoSet addresses a fundamental limitation in using multi-sample cytometry data to leverage covariates in the process of understanding the cellular correlates of particular outcomes. While CytoCoSet has demonstrated enhancements in the ability to generate representations from single-cell immune profiles that are strongly aligned with clinical outcome, through the addition of a single covariate, there could be several patient-level covariates of interest for computing embedding vectors. Therefore, as a future direction we would like to extend the CytoCoSet model to be able to include potentially many patient-level covariates, simultaneously. In line with this objective, it may also be worth considering alternative ways from the triplet penalty term to augment the loss function for inclusion of co-variate information. Overall, CytoCoSet pioneers a new direction in augmenting diverse immunophenotypes gleaned through single-cell profiling with clinical covariates to more robustly predict clinical phenotypes.

## Data Availability

The three datasets are accessible, organized, and published in Zenodo under the titles Preeclampsia (DOI: 10.5281/zenodo.10659650), Preterm (DOI: 10.5281/zenodo.10660080), and Lung Cancer (DOI: 10.5281/zenodo.10659930). The CytoCoSet software is available as open-source code on GitHub at the following URL: https://github.com/ChenCookie/cytocoset.
